# A new species of *Melitaea* from Israel, with notes on taxonomy, cytogenetics, phylogeography and interspecific hybridization in the *Melitaea persea* complex (Lepidoptera, Nymphalidae)

**DOI:** 10.3897/CompCytogen.v11i2.12370

**Published:** 2017-05-05

**Authors:** Vladimir A. Lukhtanov

**Affiliations:** 1 Department of Karyosystematics, Zoological Institute of the Russian Academy of Sciences, Universitetskaya nab. 1, St. Petersburg 199034, Russia; 2 Department of Entomology, St. Petersburg State University, Universitetskaya nab 7/9, St. Petersburg 199034, Russia; 3 McGuire Center for Lepidoptera and Biodiversity, Florida Museum of Natural History, University of Florida, 3215 Hull Rd., UF Cultural Plaza, PO Box 112710, Gainesville, Florida, 32611-2710 USA; 4 Museum of Comparative Zoology, Harvard University, 26 Oxford Street, Cambridge, Massachusetts, 02138 USA

**Keywords:** Chromosomes, *COI*, DNA barcoding, genitalia, homoploid hybrid speciation, interspecific hybridization, Middle East, *Melitaea casta*, *Melitaea eberti*, *Melitaea higginsi*, *Melitaea deserticola*, *Melitaea trivia*, morphology, nomenclature, taxonomy

## Abstract

Specimens with intermediate morphology are often considered to be the result of ongoing interspecific hybridization; however, this conclusion is difficult to prove without analysis of chromosomal and/or molecular markers. In the butterfly genus *Melitaea*, such an intermediacy can be detected in male genitalia, and is more or less regularly observed in localities where two closely related, presumably parental species are found in sympatry. Here I analyze a high altitude *Melitaea* population from Mt. Hermon in north Israel and show that its male genitalia are clearly differentiated from those found in phenotypically similar *M. persea* and *M. didyma*, but in some aspects intermediate between them. This hybrid-like population is unique because, although *M. didyma* is present on Mt. Hermon, the true, low-altitude *M. persea* has never been reported from Israel. Cytogenetic analysis revealed no apomorphic chromosomal characters to distinguish the Mt. Hermon population from other known taxa of the *M. persea* and *M. didyma* species groups. At the same time, DNA barcode-based phylogeographic study showed that this population is ancient. It was estimated to originate 1–1.6 million years ago in the Levantine refugium from a common ancestor with *M. persea*. Generally, the data obtained are incompatible with interpretation of the studied population as a taxon conspecific with *M. persea* or *M. didyma*, or a swarm of recent hybrids between *M. persea* and *M. didyma*, although the possibility of ancient homoploid hybrid speciation cannot be ruled out. I also argue that the name *Melitaea montium* assigned to butterflies from north Lebanon cannot be applied to the studied taxon from Mt. Hermon. Here I describe this morphologically and ecologically distinct entity as a new species *Melitaea acentria* **sp. n.**, and compare it with other taxa of the *M. persea* complex.

## Introduction

Butterflies of the genus *Melitaea* Fabricius, 1807 are distributed throughout the warm and temperate part of the Palaearctic region and occupy a wide range of habitat types, including meadows, grasslands, steppe, alpine biotopes, arid mountains and deserts (Tuzov and Churkin 2000). This group was revised by Higgins (1941, 1955) and more recently by Oorschot and Coutsis (2014) who used analysis of male genitalia as a main tool to document taxonomic structure of the genus. Despite these revisions, a large number of unresolved taxonomic questions persist among *Melitaea*, where species-level boundaries remain poorly defined. For example, DNA-barcode analysis revealed multiple deeply diverged lineages with properties of phylogenetic and partially biological species within *Melitaea didyma* (Esper, 1779) sensu lato, a widely distributed and common *Melitaea* species (Pazhenkova et al. 2015, Pazhenkova and Lukhtanov 2016).

Recent progress in improving our knowledge of relationships in *Melitaea* was made by using chromosomal (de Lesse 1960, Larsen 1975, Lukhtanov and Kuznetsova 1989, Hesselbarth et al. 1995) and molecular markers (Zimmermann et al. 1999, Long et al. 2014). In particular, molecular studies have helped to resolve some of the issues related to the composition of species groups within *Melitaea* (Wahlberg and Zimmermann 2000, Leneveu et al. 2009). However, with few exceptions (Kuznetsov et al. 2014, Toth et al. 2014, Pazhenkova et al. 2015, Pazhenkova and Lukhtanov 2016), molecular markers have not been used for analysis of taxonomic structure of *Melitaea* on level of closely related species or on intraspecific level.

One of the most serious problems of the *Melitaea* taxonomy is the presence of so called “intermediates” (Oorschot and Coutsis 2014). The closely related sympatric species of the genus *Melitaea* can be distinguished by male genitalia structure; however, specimens with intermediate genitalia can be more or less regularly found in nature. Most likely, these intermediates represent results of recent interspecific hybridization (Oorschot and Coutsis 2014), but such a conclusion is difficult to prove without analysis of genetic markers. The majority of these intermediates are concentrated in south-west Asia where the widely distributed species *M. persea* Kollar, 1849 contacts with *M. didyma* (in Turkey and Armenia), *M. interrupta* Kolenati, 1848 (in the Russian Caucasus, Azerbaijan, Armenia, east Turkey, Iran and Turkmenistan), *M. gina* Higgins, 1941 (in Iran) and *M. mixta* Evans, 1912 (in Afghanistan and Pakistan) (Oorschot and Coutsis 2014).

While analyzing specimens of the genus *Melitaea* collected in 2013-2016 in Israel as a part of the Israeli butterflies DNA barcoding survey project, I encountered a series of distinctive samples, collected in June 2013 at high altitude of Mt. Hermon by Asya Novikova (the Hebrew University of Jerusalem). These specimens were preliminarily identified as *M. persea montium* Belter, 1934, a name described from north Lebanon (Belter 1934) and recently established to be a synonym of *M. didyma* (Oorschot and Coutsis 2014, pages 17–18). Analysis of their male genitalia revealed them to be clearly different from phenotypically most similar *M. persea* and *M. didyma*, but in some aspects intermediate between them. A subsequent search and collecting in 2013, 2014 and 2016 resulted in a number of additional specimens from Mt. Hermon and demonstrated that this population was sympatric and partially syntopic with phenotypically similar *M. didyma liliputana* Oberthür, 1909, *M. deserticola* Oberthür, 1909 and *M. trivia syriaca* Rebel, 1905 as well as with phenotypically differentiated *M. cinxia* Linnaeus, 1758, *M. telona* Fruhstorfer, 1908 and *M. collina* Lederer, 1861.

In an effort to analyze the origin of these unusual Israeli specimens and to determine their taxonomic status, their karyotype and morphology were studied and compared to those of *M. persea* and *M. didyma*. In addition, legs were sampled from all species and major populations in the *M. didyma* and *M. persea* groups (except for the extremely rare and local *M. eberti* Koçak, 1980 from N. Iran), and sequence data from the DNA barcode region of *COI* were obtained. The results of the *M. didyma* DNA barcode survey have already been published (Pazhenkova et al. 2015, Pazhenkova and Lukhtanov 2016). Herein I present the results of the *M. persea* DNA barcode analysis, and describe the distinctive Israeli *Melitaea* as a new species, *Melitaea acentria* sp. n.

## Material and methods

### Samples

**Figure 1.**
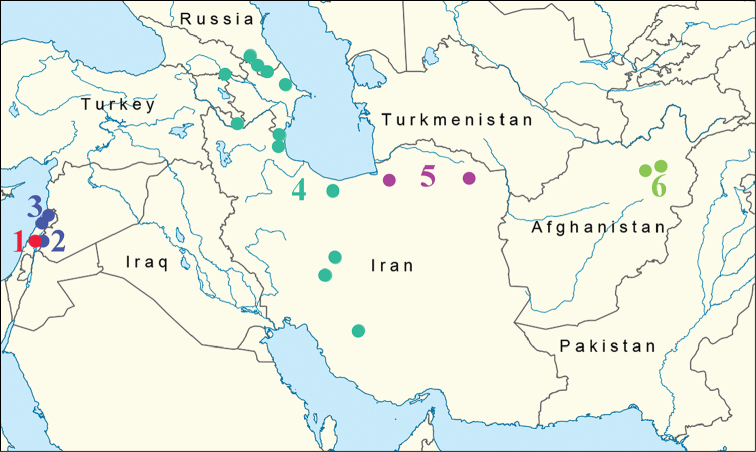
Localities from where specimens of the *M. persea* group were collected for molecular studies. **1** *M. acentria*, haplogroup A **2** *M. acentria*, haplogroup P2 **3** *M. persea*, haplogroup P2 **4** *M. persea persea*, haplogroup P1 **5** *M. persea paphlagonia*, haplogroup P3 **6** *M. higginsi*, haplogroup H.

Specimens examined are deposited in the Zoological Institute of the Russian Academy of Sciences, St. Petersburg, Russia and in the McGuire Center for Lepidoptera and Biodiversity (MGCL), Florida Museum of Natural History, University of Florida, Gainesville, Florida, USA. Photographs of all specimens used in the analysis, as well as collecting data, are available on the Barcode of Life Data System (BOLD) at http://www.boldsystems.org/. Localities where specimens of the *M. persea* group were collected are shown in Figure 1.

### Morphological analysis

For genitalia preparation, abdomens removed from adults were soaked in hot (90°C) 10% KOH for 3–10 min. Then they were transferred to water, the genitalia were carefully extracted and examined under a stereo-microscope using a pair of preparation needles or a needle and a watchmaker’s tweezer. Once cleansed of all unwanted elements they were transferred and stored in tubes with glycerine. Cleansed genitalia armatures were handled, studied and photographed while immersed in glycerine, free from pressure due to mounting, and therefore free from the ensuing distortion. Genitalia photographs were taken with a Leica M205C binocular microscope equipped with a Leica DFC495 digital camera, and processed using the Leica Application Suite, version 4.5.0 software.

The terminology of genitalia structures follows Oorschot and Coutsis (2014).

Butterfly photographs were taken with a Nikon D810 digital camera equipped with Nikon AF-S Micro Nikkor 105 mm lens.

### Molecular methods and DNA barcode-based phylogeographic study

Standard *COI* barcodes (658-bp 5’ segment of mitochondrial *cytochrome oxidase subunit I*) were studied. *COI* sequences were obtained from 92 specimens representing *M. acentria* sp. n. (25 samples), *M. persea persea* (18 samples), *M. persea* ssp. from Lebanon (2 samples), *M. persea paphlagonia* Fruhstorfer, 1917 (4 samples), *M. higginsi* Sakai, 1978 (2 samples), *M. casta* Kollar, 1849 (11 samples), *M. didyma liliputana* (7 samples), *M. deserticola* (14 samples) and *M. trivia syriaca* (9 samples) (Appendix 1: Table 1).

88 samples were processed at the Canadian Centre for DNA Barcoding (CCDB, Biodiversity Institute of Ontario, University of Guelph) using protocols described in deWaard et al. (2008), Ivanova et al. (2006) and Hajibabaei et al. (2005). Photographs of these specimens are available in the Barcode of Life Data System (BOLD) at http://www.boldsystems.org/. Legs of two samples (castaM8 and castaM9) were processed by Elena Pazhenkova at the Department of Karyosystematics of the Zoological Institute of the Russian Academy of Sciences as described earlier (Pazhenkova and Lukhtanov 2016). Two sequences, NW43-10 (http://www.nymphalidae.net/story.php?code=NW43-10) and NW43-9 (http://www.nymphalidae.net/story.php?code=NW43-9), were provided by Niklas Wahlberg. Four sequences were downloaded from GenBank: AF187796 (*M. persea*), FJ462273 (*M. persea*), FJ462238 (*M. casta*) and FJ462288 (*M. casta wiltshirei* Higgins, 1941) (Wahlberg and Zimmermann 2000, Wahlberg et al. 2005, Leneveu et al. 2009).

The barcode analysis involved 96 *COI* sequences (Appendix 1: Table 1) including 53 samples of the species close to *M. persea* (*M. persea*, *M. acentria* sp. n. and *M. higginsi*) and 13 samples of *M. casta* that was previously recovered as a sister group to *M. persea* (Leneveu et al. 2009). It also involved samples of the phenotypically similar species *M. didyma liliputana* (7 samples), *M. deserticola* (14 samples) and *M. trivia syriaca* (9 samples) collected in Israel, Jordan and Syria. Nine *M. trivia syriaca* samples were selected as an outgroup.

Sequences were aligned using the BioEdit software (Hall 1999) and edited manually. Phylogenetic hypotheses were inferred using Bayesian inference as described previously (Vershinina and Lukhtanov 2010, Lukhtanov et al. 2016a, b). Briefly, the Bayesian analysis was performed using the program MrBayes 3.2 (Ronquist et al. 2012) with default settings as suggested by Mesquite (Maddison and Maddison 2015): burn-in=0.25, nst=6 (GTR + I + G). Two runs of 10,000,000 generations with four chains (one cold and three heated) were performed. The consensus of the obtained trees was visualised using FigTree 1.3.1 (http://tree.bio.ed.ac.uk/software/figtree/).

I used two criteria to evaluate the level of DNA barcode divergence between taxa and haplogroups. First, I calculated the number of fixed DNA substitutions, i.e. the number of invariable differences in the studied *COI* fragment. Second, I calculated the minimal uncorrected *COI p*-distance between taxa and haplogroups. For this calculation, two genetically closest samples from each taxon pair were selected, and the distance between them was calculated using both fixed and non-fixed substitutions.

### Chromosomal analysis

Karyotypes were obtained from fresh adult males and processed as previously described (Lukhtanov et al. 2014, 2015a, Vishnevskaya et al. 2016). Briefly, gonads were removed from the abdomen and placed into freshly prepared fixative (3:1; 96% ethanol and glacial acetic acid) directly after capturing the butterfly in the field. Testes were stored in the fixative for 1 month at +4°C. Then the gonads were stained in 2% acetic orcein for 7–10 days at +18-20°C. Haploid chromosome numbers (n) were counted in meiotic prometaphase, metaphase I (MI) and metaphase II (MII).

## Results

### New species description

#### Melitaea acentria

Taxon classificationAnimaliaLepidopteraNymphalidae

Lukhtanov sp. n.

http://zoobank.org/A2179B2C-0B7C-4CA5-8A41-EDF93BD27D92

##### Holotype

**Figure 2.**
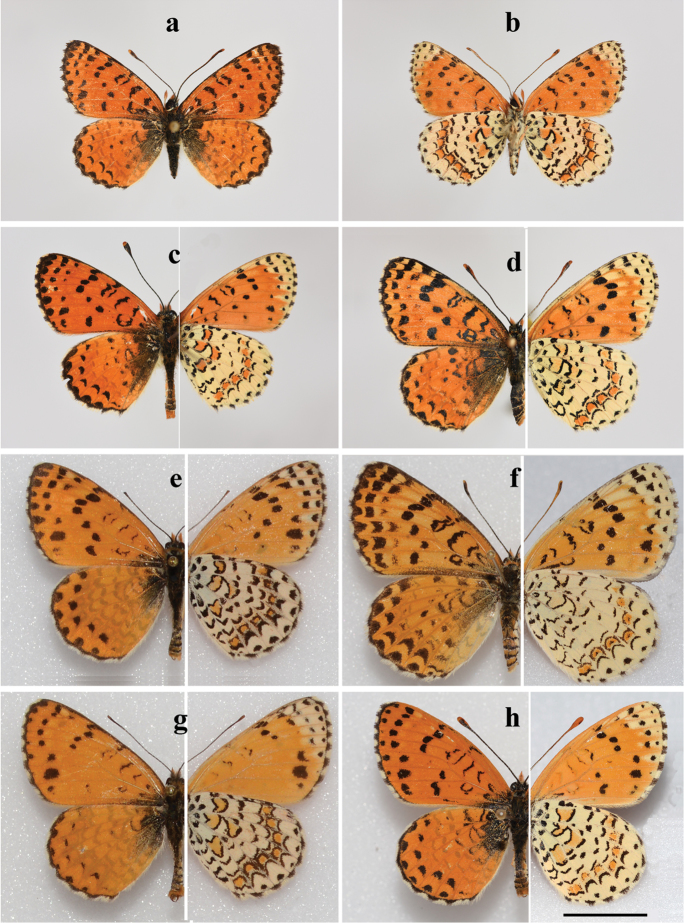
*Melitaea acentria* sp. n. and *M. persea persea*. Photos by V. Lukhtanov **a** *M. acentria* sp. n., holotype, male, sample 17949_A06, Israel, Mt. Hermon; upperside **b** *M. acentria* sp. n., holotype, male, sample 17949_A06, Israel, Mt. Hermon; underside **c** *M. acentria* sp. n., paratype, male, sample 25453_E09, Israel, Mt. Hermon **d** *M. acentria* sp. n., paratype, female, sample 25453_E11, Israel, Mt. Hermon **e** *M. persea persea*, male, 17966_A10, Iran, Fars prov., Fasa area, 20 km W Estahban, 2200 m, 9-11 May 2007, B. Denno coll., MGCL accession # 2010-20 **f** *M. persea persea*, female, 17951_B01, Iran, Fars prov., 20 km N Darab, 2100-2300 m, 24.05.1999, leg. P. Hofmann, MGCL **g** *M. persea persea*, male, 17966_A11, Iran, Fars prov., Fasa area, 20 km W Estahban, 2200 m, 9–11.05.2007, MGCL accession # 2008-43 **h** *M. persea persea*, male, 17951_B02, Iran, Char Mahall-o-Bahtiyari, Umg. Shahr-e-Kord, 2000 m, 28 May 2002, leg. P. Hofmann, MGCL. Scale bar corresponds to 10 mm in all figures.

(Fig. 2a, b), male, BOLD process ID BPAL2191-13, field # CCDB-17949_A06, GenBank accession number # KY777529; Israel, Mt. Hermon, 33°18'45.6"N; 35°47'11.9"E, 2050 m, 01 June 2013, A. Novikova leg., deposited in the Zoological Institute of the Russian Academy of Science (St. Petersburg).

*COI barcode sequence of the holotype* (BOLD process ID BPAL2191-13; GenBank accession number # KY777529): ACTTTATATTTTATCTTTGGAATTTGAGCAGGTATATTGGGAACTTCTTTAAGACTTTTAATTCGAACTGAATTAGGAA

ATCCAGGATCTTTAATTGGTGATGATCAAATTTATAATACTATTGTTACAGCTCATGCTTTTATTATAATTTTTTTTATAGT

TATACCTATTATAATTGGAGGATTTGGAAATTGATTAGTTCCTTTAATGTTAGGAGCCCCTGATATAGCATTCCCACGAATA

AATAATATAAGATTTTGATTGCTCCCCCCCTCATTAATCTTATTAATTTCTAGAAGAATTGTAGAAAATGGTGCAGGTACAG

GATGAACAGTTTACCCCCCACTTTCATCCAATATTGCTCATAGAGGATCATCTGTTGATTTAGCAATTTTTTCTCTTCATTT

AGCTGGAATTTCTTCAATTTTAGGGGCTATTAATTTTATTACCACTATTATTAACATACGCATTAATAATATATCATTCGAT

CAAATACCTTTATTTGTTTGAGCTGTAGGTATTACAGCTCTTTTATTATTATTATCTTTACCAGTTTTAGCAGGAGCAATTA

CAATACTTCTTACTGATCGAAATATTAATACTTCATTTTTTGACCCTGCTGGAGGAGGAGATCCTATTTTATACCAACATTTA

##### Paratypes.

26 males and 10 females collected on Mt. Hermon, Israel.

Four males with codes CCDB-17949_E01, BPAL2234-13; KT874736, BPAL2236-13, CCDB-17949_E03; CCDB-25452_C10, BPAL3359-16 and BPAL3360-16, CCDB-25452_C11. Two females with codes BPAL3361-16, CCDB-25452_C12; CCDB-17949_E02, KT874697, BPAL2235-13. Two females without codes. Israel, Mt. Hermon, 33°18'45.6"N; 35°47'11.9"E, 2040 m, 22 June 2013, V.A. Lukhtanov & A. Novikova leg.

Four males with codes CCDB-25453_E10, BPAL3193-16; CCDB-25453_E08, BPAL3191-16; CCDB-25453_E09, BPAL3192-16; CCDB-25454_C03, BPAL3257-16; CCDB-25453_E11, BPAL3194-16. Six males and one female without codes. Israel, Mt. Hermon, 33°18'20"N; 35°47'09"E, 2030 m, 17 May 2014, A. Novikova leg.

One male with codes CCDB-17969_A04, BPAL2759-15. Israel, Mt. Hermon, 33°18'45.6"N; 35°47'11.9"E, 2040 m, 03 July 2014, V.A. Lukhtanov & A. Novikova leg.

Ten males with codes CCDB-25458_C06, BPALB125-16; CCDB-25458_C07, BPALB126-16; CCDB-25458_C08, BPALB127-16; CCDB-25458_C09, BPALB128-16; CCDB-25458_C10, BPALB129-16; CCDB-25458_C11, BPALB130-16; CCDB-25458_C12, BPALB131-16; CCDB-25458_D01, BPALB132-16; CCDB-25458_D02, BPALB133-16; CCDB-25458_D07, BPALB138-16. One male and three females without codes. Israel, Mt. Hermon, 33°18'41"N; 35°46'49"E, 1750-1900 m, 03 May 2016, V.A. Lukhtanov & E. Pazhenkova leg.

Two females with codes 25458 E06, BPALB149-16; 25458_E08 BPALB151-16. Israel, Mt. Hermon, 33°18'51"N; 35°46'31"E, 1800 m, 07 May 2016, V.A. Lukhtanov, A. Novikova & E. Pazhenkova leg.

All paratypes are deposited in the Zoological Institute of the Russian Academy of Science (St. Petersburg).

##### Males

(Fig. 2a–c). Forewing length 16–19 mm. Forewing is roundish.

Upperside: ground color orange-red; the wing markings are small and delicate when compared to those in *M. didyma* and *M. persea*. Forewings with very narrow black marginal border fused with internervural marginal black spots. Submargimal series formed by black triangular spots on the forewings and by fine lunules on the hindwing. Forewing postdiscal series formed by small 1-3 black spots. Forewing discal series is complete or nearly complete, formed by black spots of variable size, the first four spots near costa are often enlarged. Hindwing discal series reduced or absent. Basal marking of the fore- and hindwings is delicate. Black basal suffusion is developed only near the base of hindwings. Fringe is white, checkered by black dots.

Underside: forewing ground color orange-red except for the apical part which is yellowish. Black markings delicate, reduced as compared with those of the upperside of the wing. Hindwing ground colour yellowish-white with two orange-red fascias. The red-orange submarginal fascia shows segmentation as the yellowish-white ground color spreads along the nervures. The orange-red macules of this fascia are bordered by back lunules from the outer side. From the inner side these macules are edged by black scales and additionally bordered by black lunules, giving the appearance that the proximal border of the submarginal fascia is doubly edged. Fringe white, checkered by black dots.

##### Females

**Figure 3.**
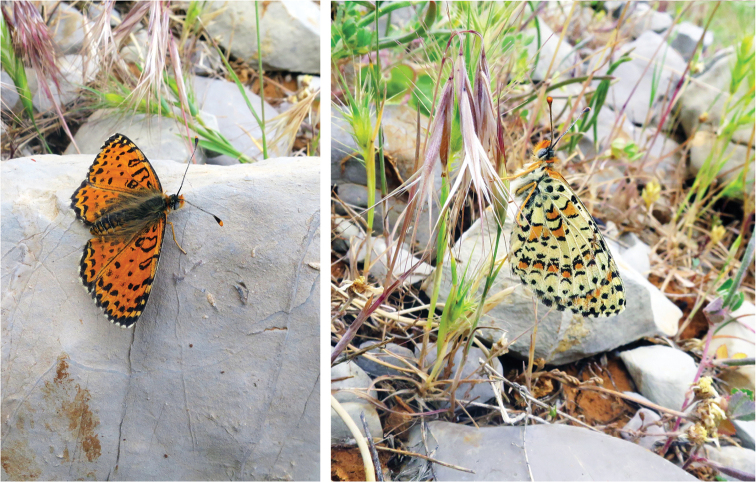
*Melitaea acentria* in nature. Female. Israel, Mt. Hermon, 1800 m, 07 May 2016. Photo by V. Lukhtanov.

(Figs 2d, 3). Forewing length 17–20 mm. Forewing is roundish. Ground color of the upperside is slightly lighter and black markings heavier than in males. Costal area of the wing apex yellow-orange. Underside of the forewings as in males but black markings are heavier and there are additional yellowish maculae between discal and postdiscal spots. Underside of the hindwings as in males. Fringe white, checkered by black dots.

##### Karyotype.

**Figure 4.**
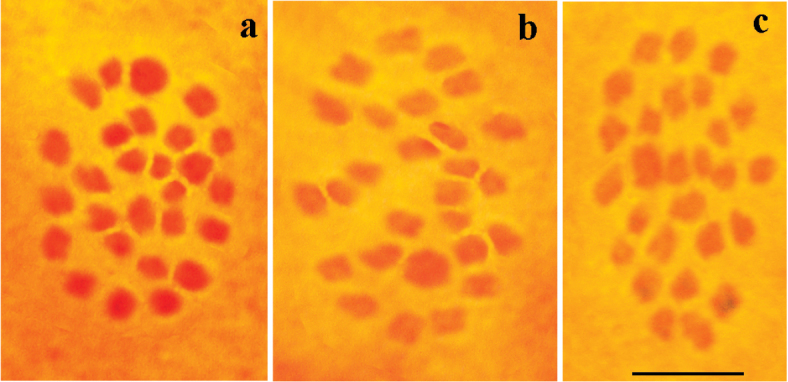
Karyotype in male meiosis of *Melitaea acentria* sp. n. from Israel. **a, b, c** sample CCDB-25458_D01, MI, n = 27. Scale bar corresponds to 5μ in all figures.

The genus *Melitaea* is known for its relatively low interspecific chromosome number variation (Pazhenkova and Lukhtanov 2016). However, in certain cases, the chromosome numbers are key to distinguish between closely related *Melitaea* species. For example, karyotype differences in combination with information about parapatric distribution were the main argument for the non-conspecificity of *M. didyma* and *M. latonigena* Eversmann, 1847 (Lukhtanov and Kuznetsova 1989). Therefore, chromosomal analysis is highly desirable in any taxonomic study of *Melitaea*. Here I conducted the chromosomal analysis of the high altitude population from Mt. Hermon (*M. acentria* sp. n.). The haploid chromosome number n=27 was found in prometaphase I, MI and MII cells of three studied individuals (2016-006, CCDB-25458_C11; 2016-008, CCDB-25458_D01; 2016-009, CCDB-25458_D02) (Fig. 4). The MI karyotype contained one chromosome bivalent that was significantly larger than the rest of the bivalents.

The same chromosome number (n=27) was previously reported for *M. persea* from Iran (de Lesse 1960). A karyotype characterized by n=27 including one large chromosome element was also found in *M. didyma neera* Fischer de Waldheim, 1840 from the North Caucasus (Russia), although in some other studied populations of *M. didyma* n=28 was found (de Lesse 1960, Lukhtanov and Kuznetsova 1989). The chromosome number n=27 was also mentioned for “*M. didyma libanotica*” from Lebanon (Larsen 1975), but the vouchers for this chromosomal analysis were larvae, and in my opinion their identification was not certain. They could represent *M. didyma liliputana*, but also *M. acentria* as well as *M. persea* (but certainly not *M. deserticola* in which n=29 was found and not *M. trivia* in which n=31 was found, Larsen 1975). Finally, n=27 was reported for “*M. montium*” from Lebanon (de Lesse 1960), but in the last case the identity of the studied samples was also not clear because the identification was not supported by genitalia analysis.

Thus, no fixed karyotype difference is known to exist between *M. acentria* and *M. persea* as well as between *M. acentria* and *M. didyma*. Therefore we cannot use the available chromosomal data for delimitation between these species.

##### Male genitalia structure.

*M. didyma* from Israel (Mt. Hermon) and *M. persea* from Iran and Azerbaijan were analyzed and were found to possess typical characters described previously (Higgins 1941, Oorschot and Coutsis 2014).

**Figure 5.**
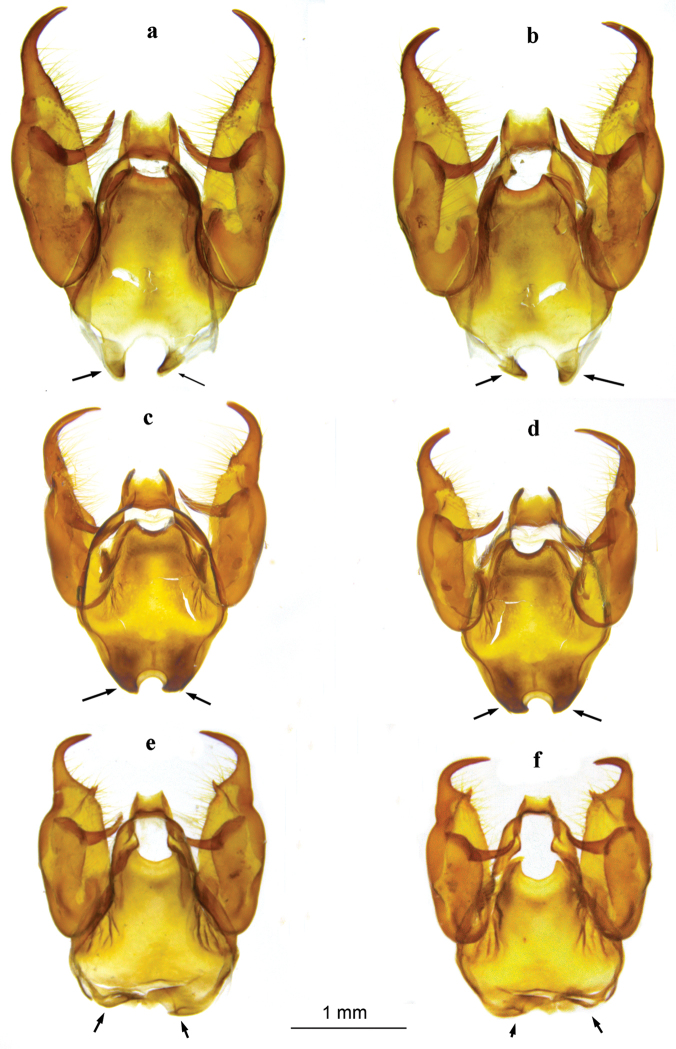
Male genitalia of *Melitaea persea persea* (sample 25450_C06, Azerbaijan, Talysh), *M. acentria* sp. n. (holotype, sample 17949_A06, Israel, Mt. Hermon) and *M. didyma liliputana* (sample 17698_E10 Israel, Mt. Hermon) (aedeagus is not shown; branches of saccus are indicated by arrows). **a** *M. persea persea*, dorsal view **b** *M. persea persea*, ventral view **c** *M. acentria* sp. n., dorsal view **d** *M. acentria* sp. n., ventral view **e** *M. didyma liliputana*, dorsal view **f** *M. didyma liliputana*, ventral view.

**Figure 6.**
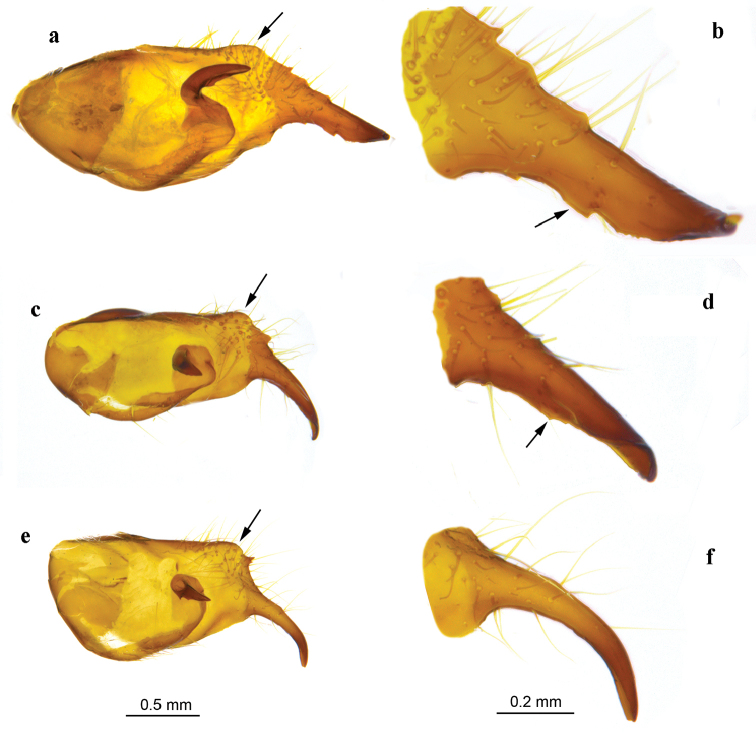
Lateral view of the inner face of right valva (left panel) and distal process (right panel). The angle between the valval dorsum and the distal process (left panel), and the keel of the distal process (right panel), are indicated by arrows. **a, b** *Melitaea persea persea* (sample 25450_C06, Azerbaijan, Talysh) **c, d** *M. acentria* sp. n. (holotype, sample 17949_A06, Israel, Mt. Hermon) **e, f** *M. didyma liliputana* (sample 17698_E10 Israel, Mt. Hermon).

**Figure 7.**
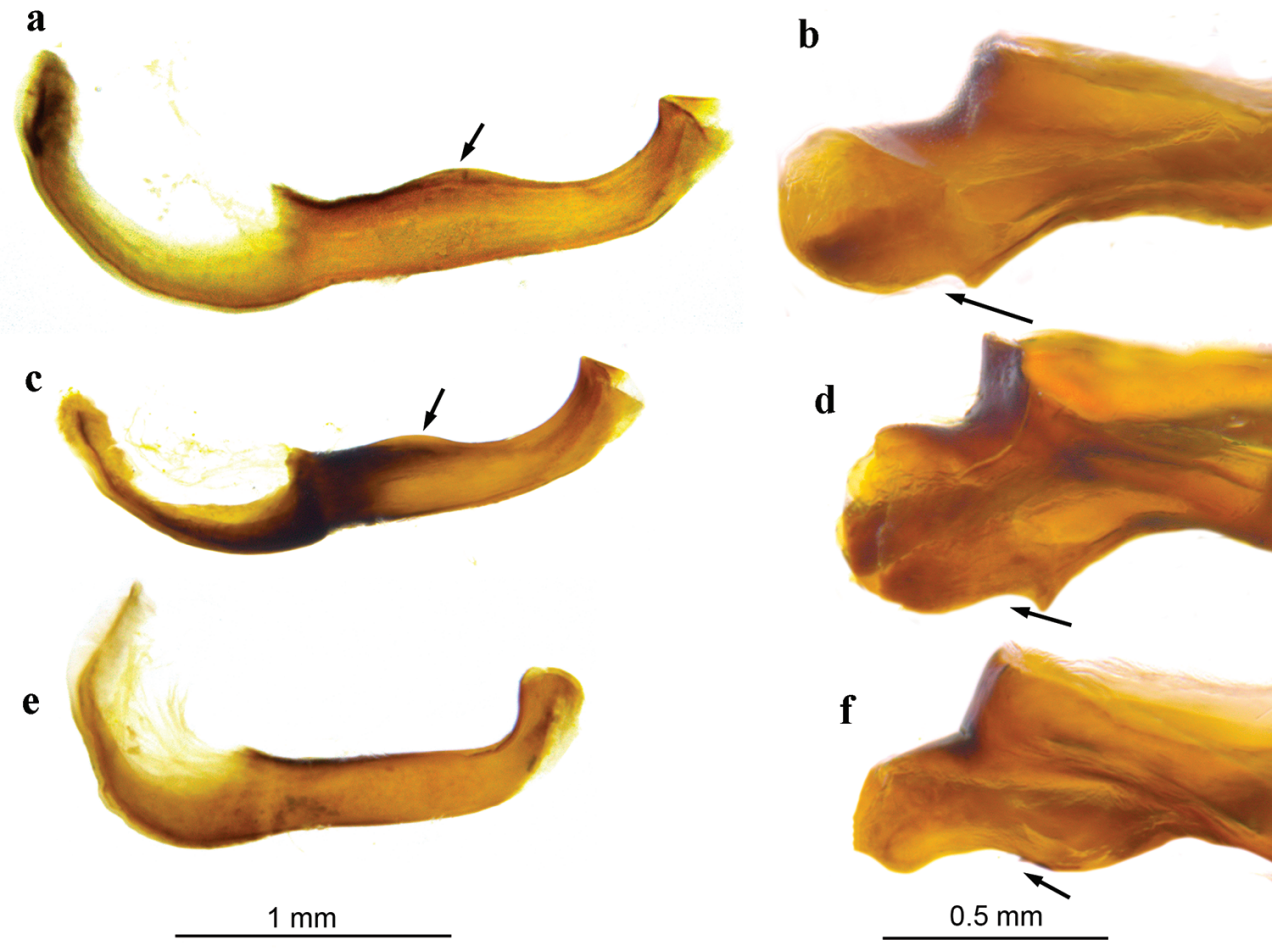
Lateral view of the left side of aedeagus and (left panel), and lateral view of the right side of tegumen (right panel). The post-zonal dorso-lateral ridge (left panel) and lateral sclerotized element (right panel) are indicated by arrows. **a, b** *Melitaea persea persea* (sample 25450_C06, Azerbaijan, Talysh) **c, d** *M. acentria* sp. n. (holotype, sample 17949_A06, Israel, Mt. Hermon) **e, f** *M. didyma liliputana* (sample sample 17698_E10 Israel, Mt. Hermon).

In *M. persea* all the main structures (ring-wall, tegumen, saccus, valvae) are elongated (Fig. 5a, b), longest in the genus *Melitaea* (Oorschot and Coutsis 2014). The valva is elongated from lateral view (Fig. 6a) and the valval distal process is massive (Fig. 6b). The dorsum of the valval distal process lies nearly in line with the remainder of the valval dorsum (Fig. 6a). The ventrum of the valval distal process possesses a keel bearing strong teeth (Fig. 6b). The saccus is bifurcate, with long, distally pointed branches (Fig. 5a, b). The aedeagus is curved, with a pronounced dorso-lateral ridge (Fig. 7a). The lateral sclerotized element of the tegumen is massive and its distal half is shaped like a smoker’s pipe (Fig. 7b).

In *M. didyma liliputana* from Mt. Hermon all the main structures (ring-wall, tegumen, saccus, valvae) are significantly shorter than in *M. persea* (Fig. 5e, f). The valva is trapezoidal from lateral view (Fig. 6e). The valval distal process is delicate (Fig. 6f) and the dorsum of the valval distal process forms a clear angle with the remainder of the valval dorsum (Fig. 6e). The ventrum of the valval distal process is smooth, without a keel and/or teeth (Fig. 6f). The saccus is bifurcate, with short, distally rounded branches (Fig. 5e, f). The aedeagus is curved, without a pronounced dorso-lateral ridge (Fig. 7e). The lateral sclerotized element of the tegumen is delicate and its distal half is T- or Γ-shaped (Fig. 7f).

**Figure 8.**
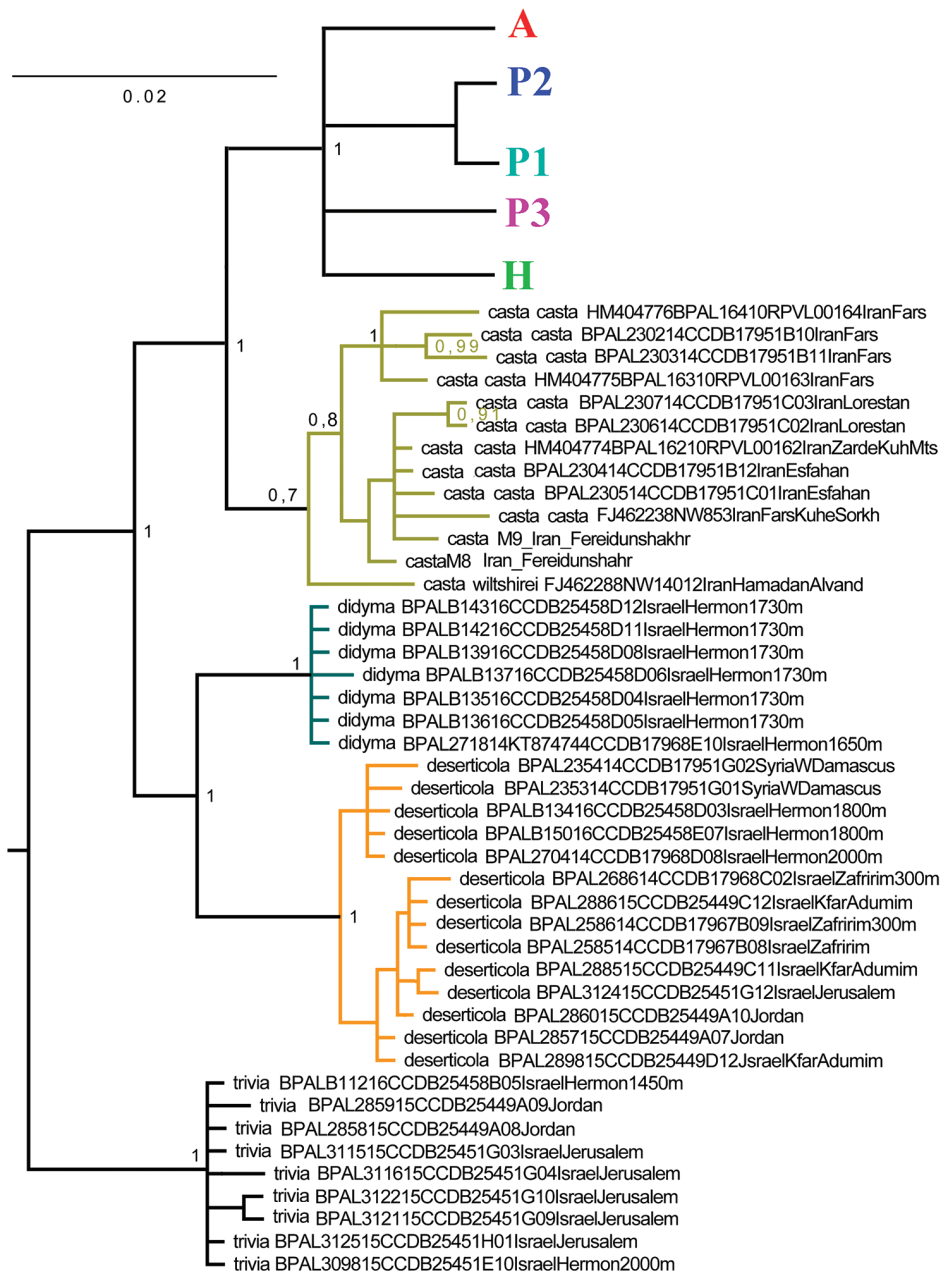
The Bayesian tree of studied *Melitaea* samples based on analysis of *the cytochrome oxidase subunit I* (*COI*) gene. Numbers at nodes indicate Bayesian posterior probability. A, H, P1, P2 and P3 are recovered haplogroups of the *M. persea* species complex (see Fig. 9 for more details).

**Figure 9.**
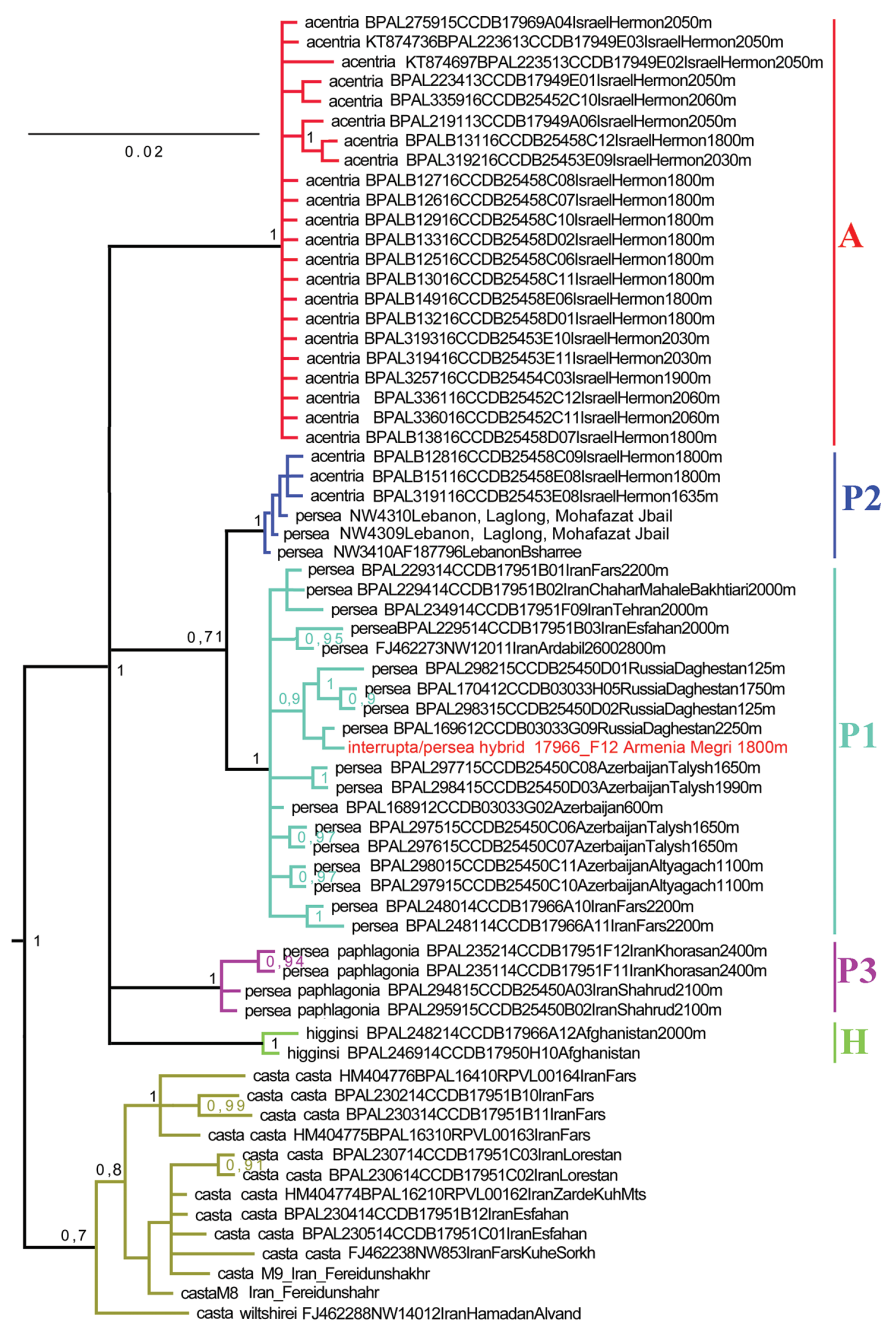
Fragment of the Bayesian tree of the studied *Melitaea* samples based on analysis of the *cytochrome oxidase subunit I* (*COI*) gene. *M. casta* and species of the *M. persea* species complex are shown. Numbers at nodes indicate Bayesian posterior probability. A, H, P1, P2 and P3 are recovered haplogroups of the *M. persea* species complex.

In *M. acentria* genitalia are clearly different from both *M. persea* and *M. didyma*, but at the same time are intermediate in some aspects. All the main structures (ring-wall, tegumen, saccus, valvae) are similar to those in *M. persea* but shorter (however, longer than in *M. didyma*) (Fig. 5c,d). The valva is cylindrical from lateral view (Fig. 6c). The valval distal process is intermediate in its shape between *M. persea* and *M. didyma* (Fig. 6c, d). Its dorsal and ventral borders are roughly parallel from lateral view (Fig. 6c). The dorsum of the valval distal process forms a clear angle with the remainder of the valval dorsum (similarly to *M. didyma*) (Fig. 6c). At the same time, the ventrum of the valval distal process possesses a keel bearing teeth (similarly to *M. persea*) (Fig. 6d). However, this keel and teeth are smaller and more delicate than in *M. persea* (Fig. 6d). The saccus is bifurcate, with relatively long, distally pointed branches; however, these branches are shorter than in *M. persea*, but longer than in *M. didyma liliputana*, where they are almost absent (Fig. 5c, d). The aedeagus is curved, with a dorso-lateral ridge (Fig. 7c); thus the aedeagus of *M. acentria* is not intermediate between *M. persea* and *M. didyma*, but similar to *M. persea*. The lateral sclerotized element of the tegumen is massive and its distal half is shaped like a smoker’s pipe (Fig. 7d). This type of male genitalia was found in all seven studied samples including two samples (25453_E08 and 25458_C09) that were characterized by the mitochondrial haplogroup P2 (Figs 8 and 9).

##### COI barcode analysis.

The *COI* barcode analysis revealed five major clusters represented by (1) *M. trivia syriaca*, (2) *M. deserticola*, (3) *M. didyma liliputana*, (4) *M. casta* and (5) taxa of the *M. persea* group (haplogroups A, H, P1, P2 and P3) (Fig. 8). Interestingly, this analysis showed that the phenotypically similar species *M. trivia syriaca*, *M. deserticola* and *M. didyma liliputana*, can be easily separated by their DNA barcodes.

The analysis recovered the *M. persea* group (*M. acentria* + *M. persea* +*M. higginsi*) as a strongly supported monophyletic clade sister to *M. casta* (Fig. 9). This clade was divided into five lineages.

The first lineage (haplogroup P1) includes a huge range of *M. persea* populations from Daghestan (Russia) in the north to Shiraz province (Iran) in the south, including samples from Shiraz in SW Iran, which represents the type locality of *M. persea*. Across this range, *M. persea* shows various degrees of localized morphological diversification, and from this territory several taxa, currently attributed to *M. persea*, were described: *M. didyma caucasica* Staudinger, 1861; *M. didyma kaschtschenkoi* Christoph, 1889; *M. didyma araratica* Verity, 1929; *M. didyma magnacasta* Verity, 1929; *Melitaea tauricus* Belter, 1934; *M. pesea hafiz* Higgins, 1941; *M. hafiz darius* Gross & Ebert, 1975 and *M. jitka* D.Weiss & Major 2000. The taxonomy of these taxa was studied in more detail by Oorschot and Coutsis (2014), who found that they are closely related and should be considered no more than synonyms of *M. persea persea*. My DNA barcode results are consistent with this conclusion (Fig. 9).

**Figure 10.**
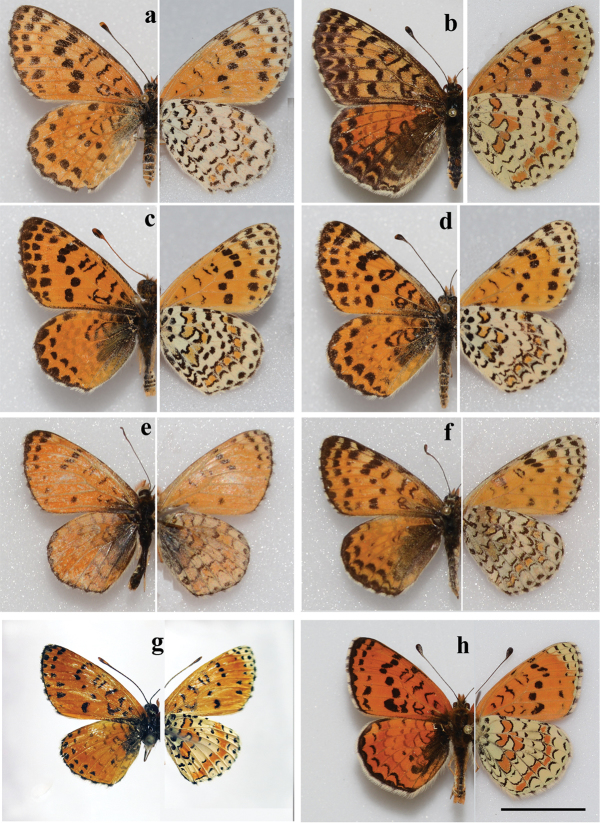
*Melitaea persea persea*, presumptive hybrid between *M. interrupta* and *M. persea*, *M. persea paphlagonia, M. higginsi*, *M. didyma liliputana* and *M. interrupta*. Photos by V. Lukhtanov **a** *Melitaea persea persea*, female, 17951_B03, Iran, Esfahan, Kuh-e-Marsenan, near Zefre, 2000 m, 26 May 2002, leg. Hofmann, MGCL **b** presumptive hybrid female between *M. interrupta* and *M. persea*, 17966_F12, Armenia, Zhangezur Range, Megri district, Litchk, 1800 m, 23 July 1999, A. Dantchenko leg., MGCL **c** *M. persea paphlagonia*, male, 17951_F11, Iran, Khorasan, Kuh-e-Binalut, 15 km SW Zoshk, 2300–2500 m, 7 June 1999, leg. P. Hofmann, MGCL **d** *M. persea paphlagonia*, male, 17951_F11, Iran, Khorasan, Kuh-e-Binalut, 15 km SW Zoshk, 2300-2500 m, 7 June 1999, leg. P. Hofmann, MGCL **e** *M. higginsi*, male, 17966_A12, Afghanistan, Hindukush, Panchir Valley, 20 June 2004, M.J.Simon collection, MGCL **f** *M. higginsi*, female, 17950_H10, Afghanistan, Badakhshan, Mt. Yamak N of Anjuman Pass, 3500-4000 m, 1-25 July 2004 M.J.Simon collection, MGCL **g** *M. didyma liliputana*, male, 17968_E10, Israel, Mt. Hermon **h** *M. interrupta*, male, 17966_F11, Armenia Armenia, Zhangezur Range, Kadjaran, 2500 m, 21–22 July 1999, leg. A. Chuvilin, MGCL; the wing underside is with black scales along the veins. Scale bar corresponds to 10 mm in all figures.

The haplogroup P1 includes also a female sample 17966_F12 possessing intermediate morphological characters between *M. interrupta* and *M. persea* (Fig. 10b). In this specimen wing upperside is similar to that in *M. interrupta*, whereas the wing underside is without black scales along the veins which are typical for *M. interrupta* (Fig. 10h), but with orange-red submarginal spots edged by black scales typical for *M. persea* (Fig. 2e–h, 10a). This sample was collected at the same place with three typical *M. interrupta* males (samples17966_F09, 17966_F10 and 17966_F11) possessing typical *M. interrupta* phenotype (Fig. 10h) and *COI* haplotypes (GenBank # KT874702, KT874740 and KT874741), which were very different from those of *M. persea* (see Fig. 4 in Pazhenkova and Lukhtanov 2016). It is thus probable that the female 17966_F12 is a result of a more or less recent hybridization between *M. interrupta* and *M. persea*. Thus, it likely represents a first molecular evidence for sporadic interspecific hybridization in *Melitaea*.

The second lineage (haplogroup P2) is represented by three specimens from north Lebanon originally identified as *M. persea* (Wahlberg et al. 2005) and by three samples of *M. acentria* from Mt. Hermon: two males (25453_E08 and 25458_C09) that were indistinguishable in their genitalia from *M. acentria* of the haplogroup A and a single female (25458_E08). This lineage was found to be closest to P1 (*Melitaea persea persea*). It differed from P1 by 7 fixed DNA substitutions in the studied 658 bp fragment of the mitochondrial *COI* gene. The minimal uncorrected *COI p*-distance between the representatives of these two haplogroups was calculated using both fixed and non-fixed substitutions and was found to be 2.0 %.

The third lineage (haplogroup P3) includes samples from NE Iran (*M. persea paphlagonia*). It differed from P1 (*M. persea persea*) by 10 fixed DNA substitutions in the studied 658 bp fragment of the mitochondrial *COI* gene. The minimal uncorrected *COI p*-distance between these two haplogroups was found to be 2.3 %. They were also distinct in wing pattern: on the upper surface all the markings were well developed and the first four spots of the discal series were nearly fused to form a prominent costal bar (Fig. 10c, d). The male genitalia of *M. persea paphlagonia* were similar to those found in *M. persea persea* (Higgins 1941). This lineage was not recognized as a taxon by Oorschot and Coutsis (2014). However, it was recognized as a distinct subspecies by Higgins (1941), and my DNA barcode results corroborate this conclusion. The level of *COI* differentiation between M. *perseapaphlagonia* and *M. persea persea* (10 fixed DNA substitutions) was found to be equal to that found between *M. persea persea* and *M. higginsi* (10 fixed DNA substitutions).

The forth lineage (haplogroup A), one of the most diverged lineages, is represented by samples from Mt. Hermon (*M. acentria*). It differed from P1 (*M. persea persea*) by 11 fixed nucleotide substitutions in the studied 658 bp fragment of the mitochondrial *COI* gene. The minimal uncorrected *COI p*-distance between these two haplogroups was found to be 2.4 %.

The fifth lineage (haplogroup H) includes samples of *M. higginsi* (Fig. 10e, f). This taxon is very rare in collections, and I have been lucky to find two specimens in the McGuire Center. It differed from P1 (*M. persea persea*) by 10 fixed DNA substitutions in the studied 658 bp fragment of the mitochondrial *COI* gene. The minimal uncorrected *COI p*-distance between these two haplogroups was found to be 2.4 %. This taxon is similar to *M. persea* with respect to male genitalia structure (Oorschot and Coutsis 2014), but quite different in wing pattern. Particularly, in males the hindwing uppersurface is without black spots which are always present in *M. persea*, and in both sexes hindwing underside veins are scaled with black, similar to *M. interrupta* and different from *M. persea*. My DNA barcode results confirm the distinctness of this high altitude very local Afghani taxon. They also confirm that this taxon is a member of the *M. persea* species group as suggested by Oorschot and Coutsis (2014), and not related to the Mongolian *M. didymina* Staudinger, 1895 as was supposed by Sakai (1978), as well as not related to *M. didyma* as was supposed by Kolesnichenko and Churkin (2004).

##### Diagnosis.

Butterfly wing pattern and male genitalia morphology, as well as DNA barcodes certaintly indicate that *Melitaea acentria* belongs to the *M. persea* species complex. After Oorschot and Coutsis (2014) this complex includes three closely related species: *M. persea*, *M. eberti* and *M. higginsi*. Male genitalia of these three species were analysed by Oorschot and Coutsis (2014) and were found to be virtually indistinguishable. *Melitaea acentria* differs from these most closely related species by several characters in male genitalia. In *M. acentria* main genitalia structures (ring-wall, tegumen, saccus, valvae) are significantly shorter. The valva is cylindrical from lateral view, not elongated (Fig. 6c). The valval distal process is intermediate in its form between *M. persea* and *M. didyma* (Fig. 6c, d). The dorsum of the valval distal process forms a clear angle with the remainder of the valval dorsum (in similar way as in *M. didyma*) (Fig. 6c), but very different from *M. persea*. The keel and teeth of the valval distal process are smaller and more delicate than in *M. persea* (Fig. 6d). On average, in *M. acentria* the ground color of the wing upperside is more orange-red (Fig. 2a–d). In other species of the *M. persea* complex it is yellowish-orange (Fig. 2e-g, 10a, c–f). However, this character is not constant (e.g. see *M. persea* with orange-red wing color on Fig. 2h). The great majority of *M. acentria* samples significantly differ from all other taxa by their DNA barcodes; however, probably due to mitochondrial introgression, a minor part of the samples cluster with the haplogroup P2 of *M. persea*.

*Melitaea acentria* significantly differs from the distantly related but phenotypically similar species *M. didyma*, *M. deserticola* and *M. trivia* by DNA barcodes and male genitalia structures. Particularly, it differs from *M. didyma* by the ventrum of the valval distal process possessing a keel bearing teeth and by the elongated shape of the ring-wall, tegumen, saccus and valvae. *Melitaea acentria* mostly differs from *M. didyma* by the hindwing underside with submarginal macules that are edged by black scales and then bordered by black lunules, giving the impression that the proximal border of the submarginal fascia is doubly edged; *M. acentria* shares this character with *M. persea*. In *M. didyma* submarginal macules of the hindwing underside are usually not edged by black scales and simply bordered by black strokes (Fig. 10g). However, elements of the black scaling of the submarginal macules can be found in few *M. didyma* samples, and sometimes this black scaling is strongly reduced in species of the *M. persea* complex.

##### Distribution.

**Figure 11.**
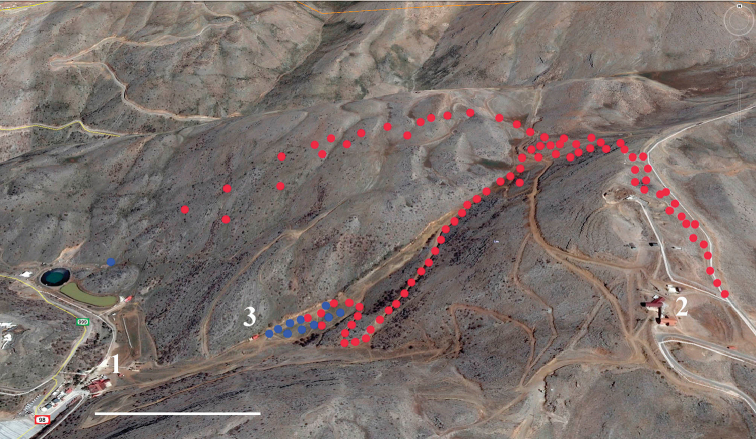
Points where *M. acentria* (red spots) and *M. didyma* (blue spots) were sampled or observed on Mt. Hermon, Israel. **1** lower station of the Hermon ski lift **2** upper station of the Hermon ski lift **3** winter café. Scale bar = 400 m.

*Melitaea acentria* is known to occur at high altitudes (1730–2060 m above the sea level) of Mt. Hermon (Fig. 11). Within these altitudes it is sympatric and syntopic with *M. trivia syriaca*, *M. deserticola* and *M. cinxia*. At the altitudes 1730–1780 m there is an essential overlapping of the *M. acentria* and *M. didyma liliputana* ranges where both species were found to fly together in early May 2016. Two other *Melitaea* species known from Mt. Hermon, *M. collina* and *M. telona*, were found to fly mostly at lower altitudes 1000–1600 m.

##### Habitat and phenology.

**Figure 12.**
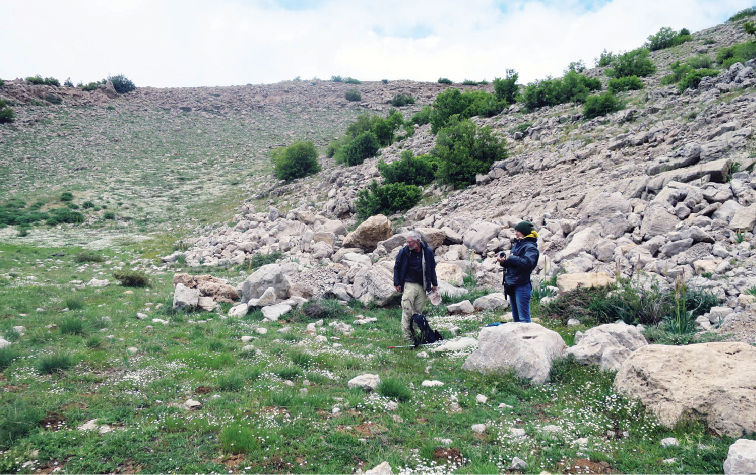
Habitat of *M. acentria*. Israel, Mt. Hermon, 1920 m, 7 May 2016. Photo by A. Novikova.

**Figure 13.**
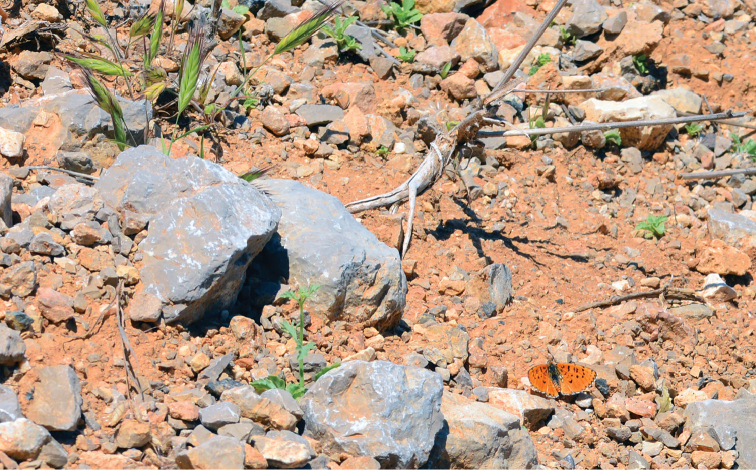
*Melitaea acentria* and its habitat. Israel, Mt. Hermon, 2040 m, 22 June 2013. Photo by V. Lukhtanov.

**Figure 14.**
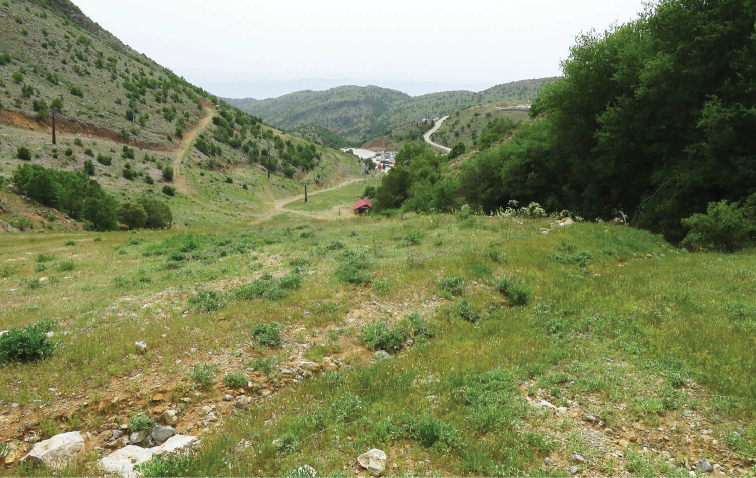
Habitat of *M. acentria* and *M. didyma liliputana.* Israel, Mt. Hermon, 1750 m, 3 May 2016. The building with red roof is the winter café shown as 3 on Figure 11. Photo by V. Lukhtanov.

**Figure 15.**
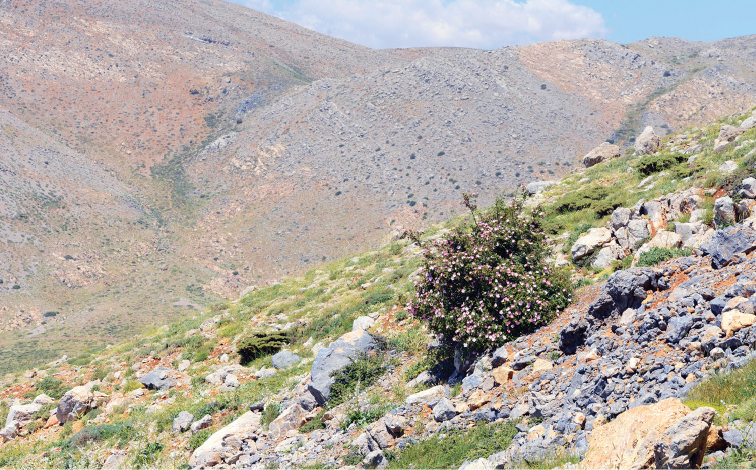
Habitat of *M. acentria*. Israel, Mt. Hermon, 2040 m, 22 June 2013. Photo by V. Lukhtanov

Three main vegetation belts have been described from Mt. Hermon: (i) evergreen Mediterranean maquis (300–1250 m); (ii) xero-montane open forest (1250–1850 m) and (iii) subalpine mountain steppe, or ‘‘Tragacanthic belt’’ (1850–2814 m) (Kent et al. 2013). Adults of *M. acentria* were found to fly in open grassy (Fig. 12) and stony (Fig. 13) areas of the upper part of the xero-montane open forest belt (1750–1850 m) (Fig. 14) and of the subalpine mountain steppe belt (1850–2060 m) (Fig. 15). Butterflies were observed from 3 May to 3 July. On the 3^rd^ of May 2016 they were abundant at altitudes from 1780 to 1900 m, therefore I conclude that they can start to fly at the end of April and continue to fly at least until mid-July.

##### Etymology.

The name *acentria* is a noun of the feminine gender. This name originates from the Greek prefix “a” that means “not” and from the Latin word “centrum” (centre) derived from the Greek “κέντρον” (kentron, a sharp point). Acentria is the Internet nickname of Asya Novikova who collected the samples initiated this research.This name indicates also the peripheral position of the new species within the distribution range of the *M. persea* species complex.

## Discussion

### Hypothesized evolutionary history of *Melitaea acentria*

*Melitaea acentria* was recovered as a diphyletic group with respect to *COI* barcodes being represented by two haplogroups A and P2. The major haplogroup A (22 samples of 25 studied) represents one of the most differentiated and thus most ancient mitochondrial lineages within the *M. persea* complex. The minor haplogroup P2 (3 samples of 25 studied) is also differentiated, but is more similar to the haplogroup P1 found in the core part of the *M. persea* species range.

To estimate the age of the haplogroup A (and the Israeli lineage as a whole) I used two calibration points: a lower rate of 1.5% uncorrected pairwise distance per million years estimated using a variety of invertebrates (Quek et al. 2004) for *COI*, and a faster rate of 2.3% uncorrected pairwise distance per million years for the entire mitochondrial genome of various arthropod taxa (Brower 1994). Using these points and the value 2.4% as the minimal uncorrected *COI p*-distance between the haplogroups A and P1, the haplogroup A can be estimated to originate approximately 1-1.6 million years ago from a common ancestor with the haplogroup P1 of *M. persea*, a species currently distributed throughout the whole Middle East. The Israeli lineage represented by haplogroup A evolved in isolation in the Levantine refugium and, most likely, relatively recently experienced episodes of hybridization with *M. persea* (haplogroup P2) resulting in mitochondrial introgression observed in the samples 25453_E08, 25458_C09 and 25458_E08. Thus, the haplogroup P2 of *M. acentria* seems to be a footprint of this introgression. Despite this supposed sporadic hybridization, the population from Mt. Hermon preserves clear diagnostic characters in male genitalia.

*Melitaea acentria* possesses male genitalia which are different from those found in both *M. persea* and *M. didyma*, but in some aspects intermediate between them. Such an intermediacy can theoretically be interpreted as a consequence of (i) an ancient hybridization resulting in homoploid hybrid speciation or (ii) a more recent hybridization resulting in the formation of a swarm of recently obtained hybrids (Lukhtanov et al. 2015b). In the first case (homoploid hybrid speciation), a new ***reproductively isolated***, sexually reproducing ***species*** arises through hybridization and combination of parts of the parental genomes, but without an increase in ploidy (Rieseberg et al. 1995, Rieseberg 1997, Coyne and Orr 2004, Lai et al. 2005). In the second case (formation of hybrid swarms), interspecific hybridization results in a number of individuals which are ***not reproductively isolated*** from their parents and, thus, do not represent a new species (Lukhtanov et al. 2015b). The second scenario caused by occasional hybridization probably occurs in *Melitaea* as demonstated by the sample 17966_F12 with an intermediate morphology and, most likely, introgressed mitochondria.

Oorschot and Coutsis (2014) treated the Lebanese samples with mixed *M. persea* - *M. didyma* genitalia type as intermediates (i.e. hybrids) between *M. persea* and *M. didyma*. Such an interpretation seems to be logical for Lebanon where *M. persea* in its more or less typical form has been reported (Higgins 1941, Larsen 1974, Racheli 1980). However, this interpretation is inappropriate for the Israeli population (i.e. for *M. acentria*). First, typical *M. persea* has never been reported from Israel, which is one of the best studied territories in the world with respect to the butterfly fauna. Second and most importantly, the *M. acentria* samples posses very divergent *COI* haplotypes which can be attributed neither to *M. persea* and nor to *M. didyma*. Thus, *M. acentria* is not a swarm of recently obtained hybrids, but an old, well-established, morphologically and ecologically differentiated lineage with clear properties of phylogenetic and biological species.

At the same time, the hypothesis that *M. acentria* is a result of ancient homoploid hybrid speciation can not be ruled out. This highly speculative hypothesis should be tested in future through full genome molecular and chromosomal studies. While such a mode of speciation is widely accepted in plants (e.g., Soltis 2013), it has only relatively recently been thoroughly investigated in animals, including butterflies (Gompert et al. 2006, Mavárez et al. 2006, Mallet 2007, Kunte et al. 2011, Dupuis and Sperling 2015, Lukhtanov et al. 2015b).

### Preimaginal stages and larval hostplant

Preimaginal stages of *Melitaea acentria* (originally identified as “*Melitaea persea montium*”) were recently described from Mt. Hermon with a comparison to the metamorphosis of *M. cinxia* (Benyamini 2016). These two species were shown to share the same larval hostplant *Plantago atrata* Hoppe, 1799 (Plantaginaceae) (Benyamini 2016).

### Why *Melitaea montium* Belter, 1934 cannot be used as a valid name?

The identity of the taxon described under the name *Melitaea montium* Belter, 1934 has never been clear. Belter (1934) reported a difference between *M. montium* and *M. didyma* in the shape of the male genitalia valva. Although this supposed difference looks very distinct in Belter’s drawing (Fig. 13 in Belter 1934) in fact it can hardly be traced. The real difference between these taxa is in the form of the tegumen, the distal process and, especially, of the saccus; however these structures were not shown on the very schematic drawings from Belter’s paper. Thus, the genitalia description and figures provide little information on the identity of the taxon described as *M. montium* (see a more detailed discussion on this topic in the monograph by Oorshot and Coutsis (2014)).

The same can be said about wing pattern. In fact, Belter was the first author who described two types of hindwing underside in *Melitaea*: (i) with the proximal double (see Fig. 2b–h; 10a, c, d, f) and (ii) with the proximal single black border of the submarginal fascia (see Fig. 10b, g, h), and mentioned that both types existed in *M. montium*. These two types were later referred to as types “a” and “b” (Hesselbarth et al. 1995). None of these two types is species-specific, although the type (i) is much more common in the *M. persea* complex, and the type (ii) is more common in the *M. didyma* complex (Hesselbarth et al. 1995, Larsen 1974).

After Belter, the name *Melitaea montium* was used in literature for the Middle Eastern (Higgins 1941, Hesselbarth et al. 1995, Tshikolovets 2011), Lebanese (Larsen 1974, Gross and Ebert 1975, Racheli 1980) and Israeli (Benyamini 2002) butterflies close or supposedly identical to *M. persea*. It was also used as a synonym of *M. didyma* (Oorshot and Coutsis 2014).

I should note that the identity of butterflies in these publications has never been clear, except for the monograph by Oorshot and Coutsis (2014) since at least three different groups of populations close to *M. persea* are recorded from the Middle East: 1) the populations close (but probably not identical) to true *M. persea* (Higgins 1941, Larsen 1974, Racheli 1980, Oorshot and Coutsis 2014), 2) *M. acentria* from Israel (this study), and 3) intermediates (hybrids) between *M. persea* and *M. didyma* from north Lebanon (Oorshot and Coutsis 2014). These three groups could be identified on basis of male genitalia characters; however, until the work of Oorshot and Coutsis (2014), the genitalia of these butterflies were not carefully studied. Higgins (1941) provided only schematic genitalia drawings that were good enough to exclude *M. didyma* from consideration, but not detailed enough to distinguish between *M. persea* and *M. persea-M. didyma* intermediates, and the consequent authors did not provide genitalia drawings at all (see the monograph of Oorshot and Coutsis (2014) for a more detailed analysis of the previous taxonomic interpretations).

The Gordian knot of this taxonomic and nomenclatural uncertainty was cut by Oorshot and Coutsis (2014) through a careful analysis of genitalia morphology, checking of all taxonomically important publications, studies of type material and subsequent designation of the lectotype of *Melitaea montium* (male, sample HO0937 in Zoologische Staatssammlung, München, collected in Lebanon, Bcharré, genitalia preparation no. 4822, figured in Oorshot and Coutsis (2014), page 188, pl. 51, figs 14, 35 and 38).

The lectotype of *Melitaea montium* was found to have typical *M. didyma* genitalia, having nothing in common with those of *M. persea*, and devoid of any intermediate characters between *M. persea* and *M. didyma* (Oorshot and Coutsis 2014, page 18). Thus, the name *Melitaea montium* represents a nominal taxon conspecific with *M. didyma*, and therefore can be synonymized with *M. didyma liliputana*, the oldest available name representing the distinct phylogenetic lineage (subspecies) distributed from north Israel, through Lebanon, Syria and east Turkey to Armenia (Pazhenkova et al. 2015, Pazhenkova and Lukhtanov 2016): Melitaea didyma race liliputana Oberthür, 1909 (Études de lépidoptérologie compare 3: 244, TL “Akbès” [SE Turkey, prov. Hatay, Akvez]) = *Melitaea montium* Belter, 1934, **syn. n.**

I should also note that, despite the valid lectotype designation resulting in this synonymy, the name *M. montium* could theoretically be preserved for a valid taxon under the plenary power of the International Commission on Zoological Nomenclature through a neotype designation. Such a possibility exists for the cases in which the existing name-bearing type of a nominal species-group taxon is not in taxonomic accord with the prevailing usage of names and stability or universality is threatened thereby (Article 75.6, http://iczn.org/iczn/index.jsp). However, in case of *M. montium* the article 75.6 can hardly be applied because de facto the prevailing usage cannot be calculated. After Belter’s publication there were few cases when this name was used, and in each case the identity of the butterflies called *M. montium* was unclear.

In this situation I see no other way than following the latest comprehensive revision (Oorshot and Coutsis 2014) that established the synonymy: *M. didyma* = *M. montium* based on lectotype designation and analysis.

## Conclusion

The *Melitaea persea* species complex consists of the following taxa:

*M. persea* Kollar, 1849

*M. persea persea* Kollar, 1849 (East Turkey, Armenia, Azerbaijan, Daghestan in Russian Caucasus, western, central and nothern parts of Iran)

*M. persea paphlagonia* Fruhstorfer, 1917 (NE Iran, probably also S. Turkmenistan)

*M. eberti* Koçak, 1980 (N. Iran)

*M. higginsi* Sakai, 1978 (Afghanistan)

*M. acentria* Lukhtanov sp. n. (Mt. Hermon in Israel, definitely also the neighboring territories of Syria and Lebanon)

The identity and taxonomic status of the *M. persea*-similar samples from north Lebanon, Jordan, Iraq, Pakistan, and Afghanistan remain still unclear. The populations from Lebanon characterized by the mitochondrial haplogroup P2 (Fig. 9) could actually represent (i) a distinct subspecies of *M. persea*, (ii) an undescribed subspecies of *M. acentria*, or even (iii) an undescribed species. Further morphological, molecular and chromosomal studies are required to select between these hypotheses.

## Supplementary Material

XML Treatment for Melitaea acentria

## Appendix

**Table 1.** List of *Melitaea* samples used in this study.

Taxon	BOLD ID	Field ID	GenBank	Locality	Reference
*M. acentria*	BPAL2759-15	CCDB-17969 A04	KY777527	Israel Hermon	This study
*M. acentria*	BPAL2236-13	CCDB-17949 E03	KT874736	Israel Hermon	Pazhenkova et al. 2015
*M. acentria*	BPAL2235-13	CCDB-17949 E02	KT874697	Israel Hermon	Pazhenkova et al. 2015
*M. acentria*	BPAL2234-13	CCDB-17949 E01	KY777528	Israel Hermon	This study
***M. acentria* Holotype**	**BPAL2191-13**	**CCDB-17949 A06**	**KY777529**	**Israel Hermon**	**This study**
*M. acentria*	BPALB127-16	CCDB-25458 C08	KY777530	Israel Hermon	This study
*M. acentria*	BPALB126-16	CCDB-25458 C07	KY777531	Israel Hermon	This study
*M. acentria*	BPALB129-16	CCDB-25458 C10	KY777532	Israel Hermon	This study
*M. acentria*	BPALB133-16	CCDB-25458 D02	KY777533	Israel Hermon	This study
*M. acentria*	BPALB131-16	CCDB-25458 C12	KY777534	Israel Hermon	This study
*M. acentria*	BPALB125-16	CCDB-25458 C06	KY777535	Israel Hermon	This study
*M. acentria*	BPALB130-16	CCDB-25458 C11	KY777536	Israel Hermon	This study
*M. acentria*	BPALB149-16	CCDB-25458 E06	KY777537	Israel Hermon	This study
*M. acentria*	BPALB132-16	CCDB-25458 D01	KY777538	Israel Hermon	This study
*M. acentria*	BPAL3193-16	CCDB-25453 E10	KY777539	Israel Hermon	This study
*M. acentria*	BPAL3194-16	CCDB-25453 E11	KY777540	Israel Hermon	This study
*M. acentria*	BPAL3257-16	CCDB-25454 C03	KY777541	Israel Hermon	This study
*M. acentria*	BPAL3361-16	CCDB-25452 C12	KY777542	Israel Hermon	This study
*M. acentria*	BPAL3360-16	CCDB-25452 C11	KY777543	Israel Hermon	This study
*M. acentria*	BPAL3359-16	CCDB-25452 C10	KY777544	Israel Hermon	This study
*M. acentria*	BPALB138-16	CCDB-25458 D07	KY777545	Israel Hermon	This study
*M. acentria*	BPAL3192-16	CCDB-25453 E09	KY777546	Israel Hermon	This study
*M. acentria*	BPALB128-16	CCDB-25458 C09	KY777574	Israel Hermon	This study
*M. acentria*	BPALB151-16	CCDB-25458	KY777575	Israel Hermon	This study
*M. acentria*	BPAL3191-16	CCDB-25453 E08	KY777576	Israel Hermon	This study
*M. casta casta*	BPAL164-10	RPVL-00164	HM404776	Iran Fars	This study
*M. casta casta*	BPAL2302-14	CCDB-17951 B10	KY777549	Iran Fars	This study
*M. casta casta*	BPAL2303-14	CCDB-17951 B11	KY777550	Iran Fars	This study
*M. casta casta*	BPAL163-10	RPVL-00163	HM404775	Iran Fars	This study
*M. casta casta*	BPAL2307-14	CCDB-17951 C03	KY777551	Iran Lorestan	This study
*M. casta casta*	BPAL2306-14	CCDB-17951 C02	KY777552	Iran Lorestan	This study
*M. casta casta*	BPAL162-10	RPVL-00162	HM404774	Iran Zarde-Kuh Mts	This study
*M. casta casta*	BPAL2304-14	CCDB-17951 B12	KY777553	Iran Esfahan	This study
*M. casta casta*	BPAL2305-14	CCDB-17951 C01	KY777554	Iran Esfahan	This study
*M. casta casta*	NW85-3		FJ462238	Iran Fars Kuh-e Sorkh	This study
*M. casta casta*	M8		KY867399	Iran, Fereidunshahr	This study
*M. casta casta*	M9		KY867400	Iran, Fereidunshahr	This study
*M. casta wiltshirei*	NW140-12		FJ462288	Iran Hamadan Alvand	Leneveu et al. 2009
*M. deserticola*	BPAL2354-14	CCDB-17951 G02	KY086108	Syria W Damascus	Pazhenkova and Lukhtanov 2016
*M. deserticola*	BPAL2353-14	CCDB-17951 G01	KY086107	Syria W Damascus	Pazhenkova and Lukhtanov 2016
*M. deserticola*	BPALB134-16	CCDB-25458 D03	KY086186	Israel Hermon 1800m	Pazhenkova and Lukhtanov 2016
*M. deserticola*	BPALB150-16	CCDB-25458 E07	KY086193	Israel Hermon 1800m	Pazhenkova and Lukhtanov 2016
*M. deserticola*	BPAL2704-14	CCDB-17968 D08	KY777564	Israel Hermon 2000m	This study
*M. deserticola*	BPAL2686-14	CCDB-17968 C02	KY777565	Israel Zafririm 300m	This study
*M. deserticola*	BPAL2857-15	CCDB-25449 A07	KY777566	Jordan	This study
*M. deserticola*	BPAL2886-15	CCDB-25449 C12	KY777567	Israel Kfar-Adumim	This study
*M. deserticola*	BPAL2885-15	CCDB-25449 C11	KY777568	Israel Kfar-Adumim	This study
*M. deserticola*	BPAL2860-15	CCDB-25449 A10	KY777569	Jordan	This study
*M. deserticola*	BPAL2898-15	CCDB-25449 D12	KY777570	Jsrael Kfar-Adumim	This study
*M. deserticola*	BPAL3124-15	CCDB-25451 G12	KY086157	Israel Jerusalem	Pazhenkova and Lukhtanov 2016
*M. deserticola*	BPAL2586-14	CCDB-17967 B09	KY777571	Israel Zafririm 300m	This study
*M. deserticola*	BPAL2585-14	CCDB-17967 B08	KY777572	Israel Zafririm	This study
*M. didyma liliputana*	BPALB143-16	CCDB-25458 D12	KY086192	Israel Hermon 1730m	Pazhenkova and Lukhtanov 2016
*M. didyma liliputana*	BPALB142-16	CCDB-25458 D11	KY086191	Israel Hermon 1730m	Pazhenkova and Lukhtanov 2016
*M. didyma liliputana*	BPALB139-16	CCDB-25458 D08	KY086190	Israel Hermon 1730m	Pazhenkova and Lukhtanov 2016
*M. didyma liliputana*	BPALB137-16	CCDB-25458 D06	KY086189	Israel Hermon 1730m	Pazhenkova and Lukhtanov 2016
*M. didyma liliputana*	BPALB135-16	CCDB-25458 D04	KY086187	Israel Hermon 1730m	Pazhenkova and Lukhtanov 2016
*M. didyma liliputana*	BPALB136-16	CCDB-25458 D05	KY086188	Hermon 1730m	Pazhenkova and Lukhtanov 2016
*M. didyma liliputana*	BPAL2718-14	CCDB-17968 E10	KT874743	Israel Hermon 1650m	Pazhenkova et al. 2015
*M. higginsi*	BPAL2482-14	CCDB-17966 A12	KY777547	Afghanistan	This study
*M. higginsi*	BPAL2469-14	CCDB-17950 H10	KY777548	Afghanistan	This study
*M. persea* (?)	NW34-10		AF187796	Lebanon Bsharree	Wahlberg and Zimmermann 2000
*M. persea* (?)	NW43-09		KY867398	Lebanon Laglong Mohafazat Jbail	This study
*M. persea* (?)	NW43-10		KY867397	Lebanon Laglong Mohafazat Jbail	This study
*M. persea paphlagonia*	BPAL2352-14	CCDB-17951 F12	KY777523	Iran Khorasan 2400m	This study
*M. persea paphlagonia*	BPAL2351-14	CCDB-17951 F11	KY777524	Iran Khorasan 2400m	This study
*M. persea paphlagonia*	BPAL2948-15	CCDB-25450 A03	KY777525	Iran Shahrud 2100m	This study
*M. persea paphlagonia*	BPAL2959-15	CCDB-25450 B02	KY777526	Iran Shahrud 2100m	This study
*M. persea persea*	BPAL2480-14	CCDB-17966 A10	KY777505	Iran Fars 2200m	This study
*M. persea persea*	BPAL2481-14	CCDB-17966 A11	KY777506	Iran Fars 2200m	This study
*M. persea persea*	BPAL2293-14	CCDB-17951 B01	KY777507	Iran Fars 2200m	This study
*M. persea persea*	BPAL2295-14	CCDB-17951 B03	KY777508	Iran Esfahan 2000m	This study
*M. persea persea*	BPAL2294-14	CCDB-17951 B02	KY777509	Iran Chahar Mahal-e Bakhtiari 2000m	This study
*M. persea persea*	BPAL2982-15	CCDB-25450 D01	KY777510	Russia Daghestan 125m	This study
*M. persea persea*	BPAL1704-12	CCDB-03033 H05	KY777511	Russia Daghestan 1750m	This study
*M. persea persea*	BPAL2983-15	CCDB-25450 D02	KY777512	Russia Daghestan 125m	This study
*M. persea persea*	BPAL1696-12	CCDB-03033 G09	KY777513	Russia Daghestan 2250m	This study
*M. persea persea*	BPAL2977-15	CCDB-25450 C08	KY777515	Azerbaijan Talysh 1650m	This study
*M. persea persea*	BPAL2984-15	CCDB-25450 D03	KY777516	Azerbaijan Talysh 1990m	This study
*M. persea persea*	BPAL1689-12	CCDB-03033 G02	KY777517	Azerbaijan 600m	This study
*M. persea persea*	BPAL2975-15	CCDB-25450 C06	KY777518	Azerbaijan Talysh 1650m	This study
*M. persea persea*	BPAL2976-15	CCDB-25450 C07	KY777519	Azerbaijan Talysh 1650m	This study
*M. persea persea*	BPAL2980-15	CCDB-25450 C11	KY777520	Azerbaijan Altyagach 1100m	This study
*M. persea persea*	BPAL2979-15	CCDB-25450 C10	KY777521	Azerbaijan Altyagach 1100m	This study
*M. persea persea*	BPAL2349-14	CCDB-17951 F09	KY777522	Iran Tehran 2000m	This study
*M. persea persea*	NW120-11		FJ462273	Iran Ardabil 2600–2800 m	Leneveu et al. 2009
*M. persea/M. interrupta* hybrid (?)	BPAL2542-14	CCDB-17966 F12	KY777514	Armenia Megri 1800m	This study
*M. trivia syriaca*	BPAL3098-15	CCDB-25451 E10	KY777555	Israel Hermon 2000m	This study
*M. trivia syriaca*	BPAL3125-15	CCDB-25451 H01	KY777556	Israel Jerusalem	This study
*M. trivia syriaca*	BPAL3122-15	CCDB-25451 G10	KY777557	Israel Jerusalem	This study
*M. trivia syriaca*	BPAL3121-15	CCDB-25451 G09	KY777558	Israel Jerusalem	This study
*M. trivia syriaca*	BPAL3116-15	CCDB-25451 G04	KY777559	Israel Jerusalem	This study
*M. trivia syriaca*	BPAL3115-15	CCDB-25451 G03	KY777560	Israel Jerusalem	This study
*M. trivia syriaca*	BPAL2858-15	CCDB-25449 A08	KY777561	Jordan	This study
*M. trivia syriaca*	BPAL2859-15	CCDB-25449 A09	KY777562	Jordan	This study
*M. trivia syriaca*	BPALB112-16	CCDB-25458 B05	KY777563	Israel Hermon 1450m	This study
